# Olfactory Cues Are Subordinate to Visual Stimuli in a Neotropical Generalist Weevil

**DOI:** 10.1371/journal.pone.0053120

**Published:** 2013-01-16

**Authors:** Fernando Otálora-Luna, Stephen L. Lapointe, Joseph C. Dickens

**Affiliations:** 1 Laboratorio de Ecología Sensorial, Centro Multidisciplinario de las Ciencias, Instituto Venezolano de Investigaciones Científicas (IVIC), Loma de Los Guamos, Parroquia Jají, Edo. Mérida, República Bolivariana de Venezuela; 2 United States Department of Agriculture, Agricultural Research Service, U. S. Horticultural Research Laboratory, Fort Pierce, Florida, United States of America; 3 United States Department of Agriculture, Agricultural Research Service, Invasive Insect Biocontrol and Behavior Laboratory, Beltsville, Maryland, United States of America; AgroParisTech, France

## Abstract

The tropical root weevil *Diaprepes abbreviatus* is a major pest of multiple crops in the Caribbean Islands and has become a serious constraint to citrus production in the United States. Recent work has identified host and conspecific volatiles that mediate host- and mate-finding by *D. abbreviatus*. The interaction of light, color, and odors has not been studied in this species. The responses of male and female *D. abbreviatus* to narrow bandwidths of visible light emitted by LEDs offered alone and in combination with olfactory stimuli were studied in a specially-designed multiple choice arena combined with a locomotion compensator. Weevils were more attracted to wavelengths close to green and yellow compared with blue or ultraviolet, but preferred red and darkness over green. Additionally, dim green light was preferred over brighter green. Adult weevils were also attracted to the odor of its citrus host + conspecifics. However, the attractiveness of citrus + conspecific odors disappeared in the presence of a green light. Photic stimulation induced males but not females to increase their speed. In the presence of light emitted by LEDs, turning speed decreased and path straightness increased, indicating that weevils tended to walk less tortuously. *Diaprepes abbreviatus* showed a hierarchy between chemo- and photo-taxis in the series of experiments presented herein, where the presence of the green light abolished upwind anemotaxis elicited by the pheromone + host plant odor. Insight into the strong responses to visual stimuli of chemically stimulated insects may be provided when the amount of information supplied by vision and olfaction is compared, as the information transmission capacity of compound eyes is estimated to be several orders of magnitude higher compared with the olfactory system. Subordination of olfactory responses by photic stimuli should be considered in the design of strategies aimed at management of such insects.

## Introduction

The tropical root weevil *Diaprepes abbreviatus* (L.1758) (Coleoptera: Curculionidae: Entiminae) is a generalist phytophagous pest that can feed on at least 270 species of plants [1]. *D. abbreviatus* has been reported as a pest within its presumed native range in Puerto Rico and the Caribbean Islands since the late 19^th^ century, feeding on economically important crops such as guava, coffee, sugarcane, lime, corn, sweet potato and cotton [2]. Since its discovery in Florida, USA in 1964, it has spread to Texas and California [3]. Now that the weevil is established in California and Louisiana, there is no geographic or climatic barrier to its movement south to Mexico, Mesoamerica and South America [3]. Larvae and pupae develop in the soil from which adults emerge to search for foliage where feeding, aggregation, mating and oviposition take place [4]. Clusters of eggs are laid and secured between mature leaves with an adhesive produced by the female thereby protecting them from dessication [5], [6]. After hatching, neonate larvae fall to the ground and enter the soil [4], thus reinitiating the life cycle. Mating occurs primarily during the day whereas females lay eggs primarily during the night [7].

Research on the sensory ecology of *D. abbreviatus* is limited. Laboratory and field observations revealed that *D. abbreviatus* is attracted to odors emanating from citrus foliage, green beans, conspecifics and conspecific feces (i.e., frass) [8], [9], [10], [11]. In one study, preference by *D. abbreviatus* for food odors was influenced by prior feeding [10]. The presence of a male-produced aggregation pheromone was postulated previously [7], [9], [11] based on behavioral observations. Specific host plant volatiles involved in the orientation behavior of *D. abbreviatus* were identified [12] and recently an aggregation pheromone was characterized from the headspace of D. *abbreviatus* males feeding on citrus [13]. This unsaturated hydroxy-ester, recently identified as methyl (E)-3-(2-hydroxyethyl)-4-methyl-2-pentenoate, elicited behavioral responses in a two-choice olfactometer. This pheromone, alone or in combination with plant volatiles, may play a role in long-range location, as it has a low electrophysiological antennal sensory threshold and is produced by clusters of males observed in the field on so-called “party trees” [13].

Less attention has been paid to the response of weevils to visual stimuli. Peak spectral sensitivity of the compound eye of *D. abbreviatus* was demonstrated at 530 nm (green) and a small number of adults were captured at night with traps equipped with a fluorescent green light [14]. However, most studies of behavioral responses of arthropods to light have used pigmented surfaces which reflect a range of wavelengths. Colors emitted by light emitting diodes (LEDs) do not depend on the illuminating conditions and allow for selection and comparison of spectra of experimental light sources. Recently, a multiple choice arena adapted to a servosphere was developed to observe walking of Colorado potato beetle [*Leptinotarsa decemlineata* (Say)] responding to colored LEDs [16].

Although the interaction of vision and olfaction in *D. abbreviatus* orientation has not been studied, several authors have investigated multimodal stimulation in other insects [17], [18], [19], [20], [21], [22], [23], [24]. Here we investigated the response of male and female *D. abbreviatus* to narrow bandwidths of visible light emitted by LEDs offered alone and in combination with olfactory stimuli. Our results provide insight into the mechanisms involved in the response of a polyphagous weevil, and probably other insects, to multimodal sensory inputs and reveal that the response to chemical attractants can be modulated by visual stimuli.

## Materials and Methods

### Insects

Newly emerged virgin adult *D. abbreviatus* were obtained from the U.S. Horticultural Research Laboratory, Fort Pierce, FL, USA [4], [25]. Adults were identified by sex, and shipped overnight in individual plastic cups with a piece of moist dental wick to the Invasive Insect Biocontrol and Behavior Laboratory, Beltsville, MD, USA where the experiments were performed. Females and males were kept separately in groups of 20 in round plastic containers (height 25 cm, diameter 19 cm) in an environmental chamber at 25±1°C and 80±5% relative humidity. Adults were provided young leaves of *Citrus macrophylla* Wester until use. Illumination during the photophase (12 L:12 D) was provided by a fluorescent lamp (FG12180-A, General Electric, USA). Weevils tested between the second and fourth week after emergence were starved for approximately 24 hours prior to behavioral experiments. A 2 mm square of adhesive tape was placed on the elytra of weevils used in behavioral assays to prevent flight. Individuals were used only once.

### Servosphere

**Figure 1:**
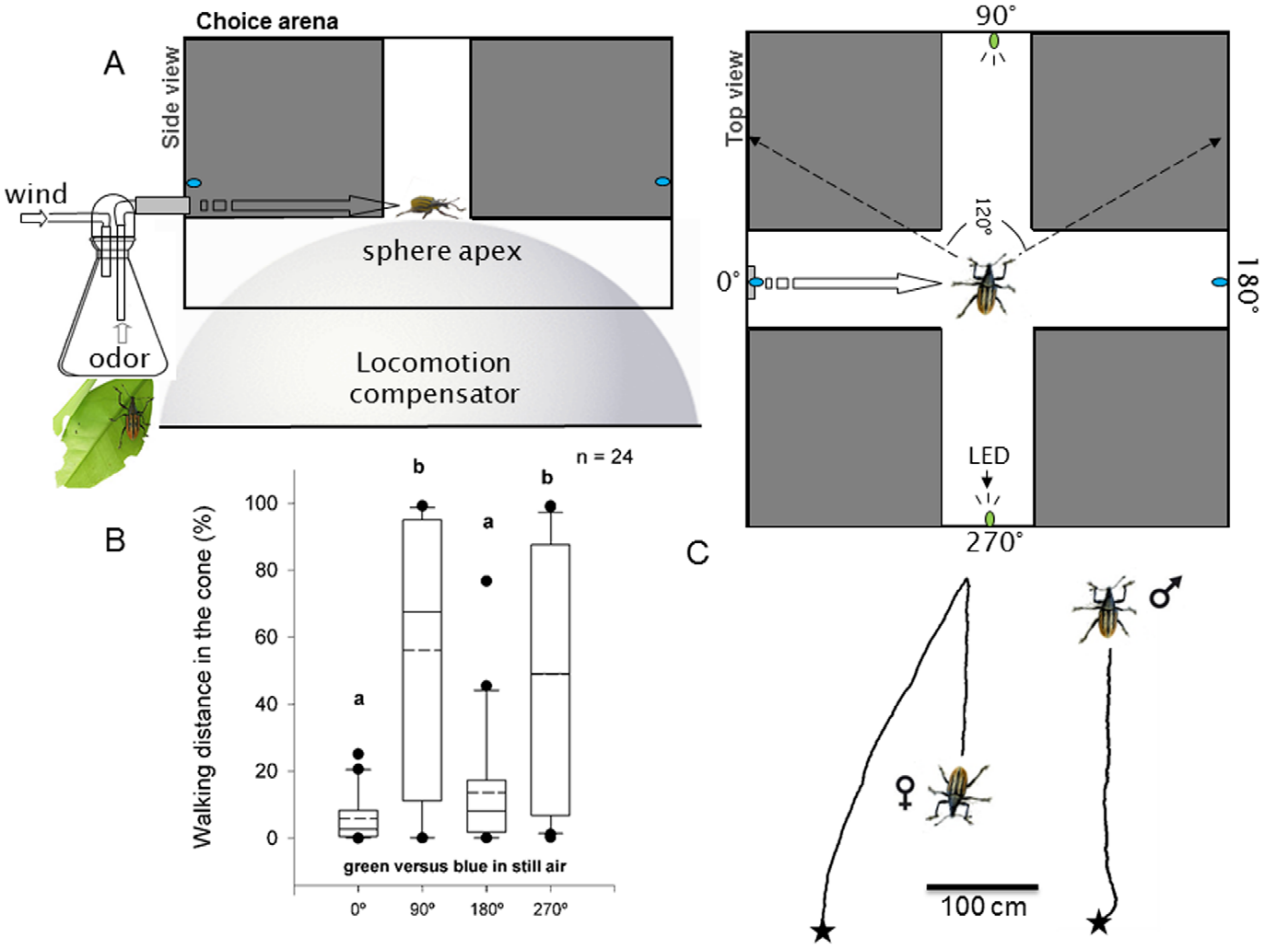
Choice arena adapted to a locomotion compensator (servosphere). Schematic representation of a choice arena adapted to a servosphere where a *Diaprepes abbreviatus* weevil walking on the apex of the sphere was stimulated with light emitting diodes (LED) of different wavelengths and chemical stimuli. (A) The sphere compensated for the insect displacement so it remained on its apex (side view). Air passing through a bottle, that contains citrus or males feeding on citrus, carried odors to the weevil. Two different colored lights were presented in paired combinations with LEDs of the same color cue positioned in opposite arms (top view). (B) Walking response of *D. abbreviatus* adults exposed to colored LEDs in still air expressed as percentage of distance covered in each of four 90° angles projected to 0°, 90°, 180° and 270° where 0° and 180° represent blue and 90° and 270° represent green. Relative displacement in the ‘x’ and ‘y’ corridors for this experiment is also showed in figure 2. Different letters indicate significant pairwise differences across the different directions (α = 0.05, Dunn’s post-hoc test). In the boxplots, bold lines indicate medians, segmented line indicate the means, lower and upper boundaries of a box indicate the 25 and 75% quartiles, respectively, whiskers below and above the box indicate the 10th and 90th percentiles, respectively, and circles represent data beyond these limits (outliers). (C) Walking trajectories performed by a female (left) and a male (right) weevil stimulated with green and blue on the servosphere showed that both individuals oriented toward the green lights. Relative size of insects has been exaggerated for clarity.

*D. abbreviatus* were tracked while walking on a servosphere (locomotion compensator) (Syntech LC-300, Hilversum, The Netherlands) [26], [27]. This tracking instrument allowed the untethered insect to walk unimpeded in all directions on the apex of a 30 cm diameter white sphere (Fig. 1). A movement detector based on active pixel sensor (APS) technology integrated with a near-infrared 8-LED (light-emitting diode) lamp (wavelength peak at 940 nm) was positioned 22 cm above the insect. The near-infrared lamp was integrated with the sensor and illuminated a field of 7 cm in diameter on the apex of the sphere. The signal from the movement detector was processed and sent to servo-motors that drove the sphere in the opposite direction of the insect’s movement in order to maintain its location at the apex of the sphere. Information on movements of the sphere was supplied to a computer by two pulse-generator encoders positioned orthogonally at the equator of the ball, thus allowing for reconstruction of tracks described by the insect’s movements. The movements were measured at a rate of 0.1/s with an accuracy of 0.1 mm.

### Multiple Choice Arena

The observation system used in this study was tested previously with *L. decemlineata* [16], [24] and consisted of a modified version of a crossroad arena [28]. It comprised two perpendicular corridors (length 36 cm, wide 7 cm, height 14 cm) made of acrylic plastic (polymethyl methacrylate) walls (thick 5 mm) (Precision Plastics Inc, Beltsville, MD, USA) fitted over a servosphere but allowing the sphere to rotate freely underneath the corridors (Fig. 1). The two corridors bisected each other forming a cross with a square chamber at the apex of the servosphere which coincided with the field of view of the video sensor. The walking insect always rested within this chamber where it was exposed to the experimental stimuli but was unable to reach any of the four arms due to the motion compensation of the servosphere (open loop set up). The inner lateral walls of the arena corridors were covered with steel metal mirrors to avoid biases caused by shadows produced at the corners of the corridors. The top of each arm was covered with a black roof made of acrylic plastic. The central chamber top was left uncovered in order to track the insect movement with the video sensor and to allow odors to exit the arena (see Chemical stimuli).

### Photic Stimuli

Within the multiple choice arena, different pairs of monochromatic lights produced by light emitting diodes (LEDs, RadioShack, Fort Worth, TX, USA; LED Supply, Randolph, VT, USA) were offered during a two minute test period (Fig. 1). The optical axes of the light beams were directed towards the insect that walked on the apex of the sphere by positioning LEDs at an angle of 9° to the insect at the end of each of the four corridor arms (Fig. 1). LEDs were the only source of visible light in the experimental room, so they were perceived by the insect as spots of light in the dark. Intensity and wavelength of emitted light were measured with a spectrometer (USB4000; Ocean Optics, Dunedin, FL, USA). The intensity of light emitted by each LED was regulated with a stimulator device (Grass S44 and S88; Grass Instruments, Quincy, MA, USA), so each LED emitted a photon intensity or flux [29] of approximately 1.55×10^17^ photons m^−2^ s^−1^. Green LEDs for low intensity light experiments emitted approximately 1.55×10^10^ photons m^−2^ s^−1^ (dim green). Colors emitted by LEDs had narrow wavelength bands that varied in width from 50 to 90 nm; these spectral bandwidth ranges were calculated from the two wavelengths on either side of the spectrum given at the intensity value that equals ½ of the peak value. The beam radiation pattern corresponded to a narrow-angled specular LED. Maximum peaks and centroid wavelengths did not differ by more than 5 nm, i.e., wavelength shapes were highly symmetric. Peak wavelengths tested occurred at 351 (ultraviolet), 472 (blue), 570 (green), 585 (yellow) and 660 nm (red) [16]. Color names correspond to the subjective visual sensation produced on the human eye by these wavelengths. Corridors not supplied with LEDs were dark.

Opposite arms were equipped with the same color cue at the end of each corridor to provide a symmetrical photic environment. Four stimulation points were used rather than two to produce two uniform fields of vision. Two LEDs would each produce two different stimuli differing in shape and intensity: the point source and the reflected light on the opposing wall. The insect would see the light emitted directly by a particular color LED, and the light reflected on the opposite wall of the arena that appears as a different shape and intensity. By using 4 LEDs (two opposite LEDs for each color in the perpendicular corridors), the insect was presented with two homogeneous sets of stimuli. The arrangement of corridors avoided reflection by the walls of the arena and the blending of the different colors being tested. The use of corridors reduced sources of reflected light, i.e., each corridor (‘x’ and ‘y’) channeled and separated the light reaching the insect from that reflected by the surroundings.

### Chemical Stimuli

Headspace odors were provided by 2.4 g of *C. macrophylla* leaves or 10 male *D. abbreviatus* feeding on similarly prepared *C. macrophylla* leaves placed in 500 ml glass gas-wash bottles (Fig. 1). Gas-wash bottles were left to equilibrate for at least 10 min before presenting volatiles in the headspace as the odor stimulus to the weevils. Insects were stimulated during a two-minute test period (see below). The odor delivery system consisted of a charcoal-filtered air-stream maintained at 25°C and 60–73% r.h. as measured by a hygro-thermometer (EA25, Extech Instruments, Waltham, MA, USA; accuracy: ± 3% r.h. and ± 1°C) flowing at 6 l/min (30 ± 15 cm/s airspeed as measured by a hot-wire anemometer, model 441S, Kurtz, CA, USA; accuracy ± 0.01 m/s) that passed through a stainless steel tube (dia. 2 cm, length 4 cm). This tube was painted black, to exclude unexpected influences from asymmetries, and positioned on the horizontal plane tangent to the apex of the sphere, i.e., at an angle of 0° to the insect, and 18 cm from the insect at the end of one corridor of the multiple choice arena. A laminar flow over the insect was made by passing the airflow through a spongy steel insert within the tube. A second charcoal-filtered air-stream (Stimulus Controller CS-55, Syntech, The Netherlands) passing through the gas-wash bottle containing the odor sources was injected at 150 ml/min immediately downstream of the spongy steel, so the air from the flasks was diluted 40-fold in the main air stream. Note that this second air flow was set in order to assure that the stimulus was constant (i.e., not depleted) during the 2 minutes period of stimulation. During the chemo-stimulation period 300 ml out of 500 ml were displaced from the odor bottle. Additionally, > 15 minutes elapsed between experiments so insects and plants had time to emit more volatiles and bottles with odors were replaced after testing 10 insects. The control consisted of a clean gas-wash bottle; the headspace of this bottle was injected during the control periods through the second charcoal-filtered air-stream. The onset and cessation of the light stimulus to the insect was synchronized with that of the chemical stimulus by use of a solenoid valve. The experimental setup was positioned near an exhaust hood to remove odors from the laboratory arena.

### Behavioral Experiments

Two sets of experiments were performed. One set consisted of experiments in which each insect was given a choice between two monochromatic LED pairs in still air (group 1). Responses of weevils to colored lights were recorded for 3 min [16]. The other set of experiments comprised 4 series of bioassays (group 2). In the first series, weevils were stimulated during the test period with air that had passed over the headspace of citrus leaves (series I: citrus alone) and in the second series, weevils were stimulated with air that had passed over male weevils feeding on citrus leaves (series II: citrus + males). In a third experimental series, weevils were stimulated with colored LEDs during the test period; a blue LED was placed above the tube that served as source of clean air that stimulated the insect and another blue LED was placed on the opposite arm of this corridor, while the other two arms were provided with a green LED each (series III: clean air + blue vs. green). Finally, the insect was simultaneously subjected to chemical and photic stimuli (series IV: citrus + males + blue vs. green) during the test period. In this series, each insect was given the choice between a blue LED + volatiles from males feeding on citrus versus another opposite blue LED and two green LEDs; blue and green LEDs were positioned as in series III. In all experiments, LEDs were the only source of illumination in the room. Responses were recorded for three consecutive 2-min periods [24]: in the air stream alone (control), in the air stream plus other experimental stimuli (test), and in the air stream after removal of the experimental stimuli (end-control). The eolic stimulation (air stream) was supplied during control, test, and end-control periods, but chemical and photic stimuli were only supplied during the test period. Experiments were performed during insect photophase, in a dark room at 25±0.5°C and 40±10% RH as measured by a hygro-thermometer (EA25, Extech Instruments, Waltham, MA, USA, accuracy: ± 3% r.h. and ± 1°C). To control for unexpected influences from asymmetries or bias within the apparatus and room, the colors were changed between the corridors after testing half of the insects. Each of the four experimental series was run with both females and males. As *D. abbreviatus* can be reluctant to move for several hours after being manipulated, most weevils were induced to walk by touching the tarsus of a metathoracic leg with forceps for less than one second. Insects manipulated in such a manner often walked continuously on the servosphere for hours. After this evasive response, each insect was allowed to adapt to the servosphere by walking during ≥5 min before starting a recording to allow it to acclimate to the experimental conditions, i.e. diminish any stress caused by the experimental procedures. Insects that failed to walk were discarded (approximately 10%).

### Track Analysis

The x-y coordinates provided by the servosphere at intervals of 0.1 s were merged in step-sizes of 10 units for more efficient summarizing of the tracks [30], [31]. This merger provided step-size intervals of 1 s which allowed the insect to move at least 50% of its length before recording its next position. This merger of intervals took into account that the mean length of *D. abbreviatus* reared with F1675 artificial diet was approximately 20 mm [25] and its average speed was approximately 20 mm/s. This step-size was large enough to reduce noise produced by insect movements such as wobbling while walking. Instantaneous displacement and direction were computed from the position changes within each interval. Kinematic parameters such as speed (i.e., magnitude of velocity), linearity, path straightness and turning velocity were calculated from instantaneous values using previously developed equations [32], [33]. Linearity was calculated by dividing displacement (distance in a straight line from origin to end point) and walking distance. Path straightness was calculated as the length of the circular mean vector that describes the weevil’s path. Both linearity and path straightness served to measure tortuosity of the path. Walking tracks were reconstructed by plotting the cumulative addition of consecutive positions. To identify stops, 3 mm/s was considered the minimum speed *D. abbreviatus* had to achieve to be considered walking; values below this speed were produced by other movements such as grooming the antennae or legs. Speed, turning speed and path straightness indices for each series were calculated as each insect’s average parameter during the test period minus its average parameter during the control period.

### Characterization of Weevil Orientation

Two methods were used to characterize weevil orientation responses according to the number of choices offered to each individual in order to facilitate presentation of the data. Relative displacement in the ‘x’ and ‘y’ corridors was compared to evaluate response during 3 minute runs when offered the choice of two pairs of colored LED lights. This system allowed for comparison of all experiments where colors were evaluated. To characterize the effects of chemical and photic stimuli on weevil orientation (i.e., four choices) each arm within the arena was assigned with an arbitrary angle of 45° either side of each stimulus. In the “multimodal experiments” where more than two choices were offered, we considered that a weevil chose a particular corridor (0°, 90°, 180° or 270° directions) when it walked into the corresponding area described by the cardinal direction ± 30°. Percentage of displacement within cones defined by these angles was calculated for each 2-min track (control, test and end-control periods).

### Statistics

Equality of variance was tested with Levene’s test and normality of the residuals of models was analyzed within each treatment with Shapiro-Wilk’s W test Shapiro-Wilk and QQ normal plot method [34], [35]. When residuals of our model followed a normal distribution and showed homoscedasticity, they were analyzed with ANOVA and t-test; otherwise, they were analyzed with Kruskal-Wallis, Wilcoxon (paired and unpaired) and Dunn’s post-hoc tests (α = 0.05). Interquartile range (IQR) was used as a measure of statistical dispersion when residuals did not follow a normal distribution, being equal to the difference between the upper and lower quartiles. The effect of stimulation on orientation and kinematic responses was also evaluated by using indices representing the difference between the data obtained for the test and the previous control period for each treatment. Post-hoc multiple comparisons were done when a Kruskal–Wallis test was significant [36]. Data were combined when females and males did not differ in behavioral responses. Results were considered significant at the 5% level; significance levels (*P*) were given in approximate values to facilitate reader comparisons. Statistical analyses were performed using R software version 2.12.2 (2011-02-25), Vienna, Austria [37].

## Results

### Orientation Responses to Colored Lights in Still Air Conditions

**Figure 2:**
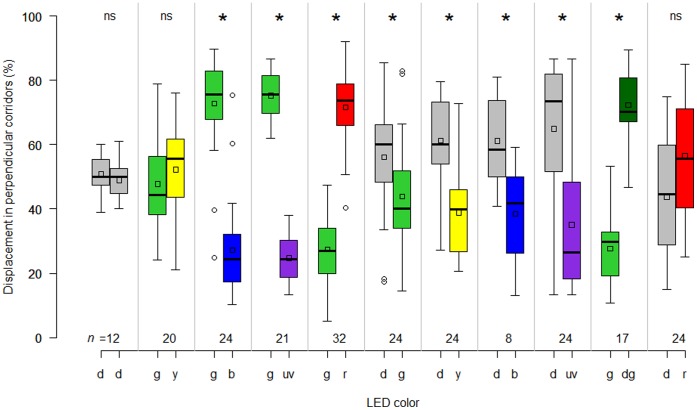
Orientation of weevils to colored lights in still air. Walking response of *Diaprepes abbreviatus* adults exposed to colored LEDs in a multiple choice arena adapted to a servosphere expressed as percent displacement in perpendicular corridors. Relative displacement in the ‘x’ and ‘y’ corridors was compared to evaluate response during 3 minute runs when offered the choice between two different pairs of colored LED lights. Asterisks indicate significance (Wilcoxon signed rank test, α = 0.05), ‘ns’ is not significant and numbers on the horizontal axis are sample size, d = dark, y = yellow, g = green, b = blue, uv = ultraviolet, r = red, dg = dim green. Note that these experiments were performed in still air. In the boxplots, bold lines indicate medians, squares indicate the means, lower and upper boundaries of a box indicate the 25 and 75% quartiles, respectively, whiskers below and above the box indicate the 10th and 90th percentiles, respectively, and circles represent data beyond these limits (outliers).

Visual choices (group 1) presented to female and male weevils in still air elicited different orientation responses (Kruskal-Wallis test, *P*<0.001) (Fig. 2). In complete darkness, there was no preference for either corridor (Wilcoxon paired test, *P* = 0.087). When green and yellow colors were offered simultaneously female and males did not show a preference (Wilcoxon paired test, *P* = 0.693). Weevils walked more towards the green light when provided a choice between green and blue during the two-minute tests (Wilcoxon paired test, *P* <0.001) and also toward green when provided a choice between green and UV (*P*<0.001). However, weevils preferred red over green (*P*<0.001). When green lights and a dark corridor were presented, insects preferred to orient their walk towards the darkened corridors (Wilcoxon paired test, *P*<0.01), and when green lights were presented vs. low intensity (i.e., dim) green lights, the later were preferred (Wilcoxon paired test, *P*<0.001). Finally, weevils showed no preference between red and darkness (Wilcoxon paired test, *P* = 0.477).

### Orientation Responses to Volatiles

**Figure 3:**
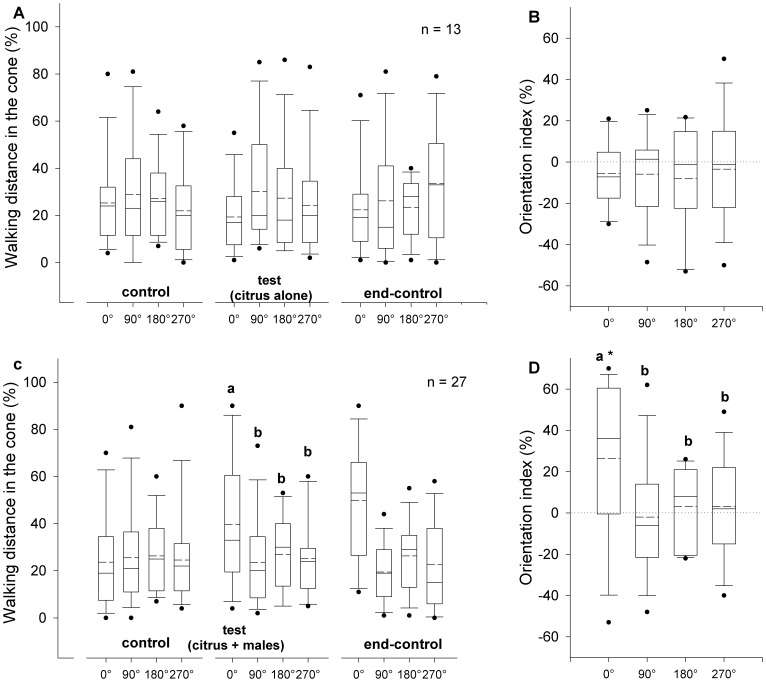
Orientation of weevils to citrus volatiles or volatiles produced by males feeding on citrus. Orientation responses of *Diaprepes abbreviatus* adults, recorded during three consecutive 2-minute periods: **A.** clean air (control), air passing through headspace of citrus foliage (test) and clean air again (end-control), or **C.** control, air passing through headspace of 10 male weevils feeding on citrus foliage (test) and end-control, were expressed as percentage of distance covered in each of four 90° angles projected to 0°, 90°, 180° and 270°. Orientation indices (**B.** and **D.** respectively) were calculated as the proportion of distance covered during the test period minus the proportion of the distance covered during the control period in each angle. Different letters indicate significant pairwise differences across the different directions (α = 0.05, Dunn’s post-hoc test). Asterisk indicates significance (α = 0.05, Wilcoxon signed rank test). In the boxplots, bold lines indicate medians, segmented line indicate the means, lower and upper boundaries of a box indicate the 25 and 75% quartiles, respectively, whiskers below and above the box indicate the 10th and 90th percentiles, respectively, and circles represent data beyond these limits (outliers).

In the absence of light, the addition of citrus volatiles to the air stream (series I) during the test period did not elicit an orientation response (Fig. 3A, B). Percentages of walking distance in the 90° angles assigned to each of the four arena arms were not significantly different between the four directions during the control, test or end-control periods (Kruskal-Wallis test, α = 0.05) (Fig. 3A). The distance walked within each angle as a percent of the total distance did not differ between the test and the previous control period, i.e., indices were not significantly different from zero (Wilcoxon signed rank test, *P* = 0.37) (Fig. 3B). Orientation indices were not significantly different between the four directions (Kruskal-Wallis test, α = 0.05). However, the addition of volatiles produced by 10 males feeding on citrus foliage to the air stream in the absence of light (series II) induced weevils to walk more upwind (0°) towards the odor source compared with either crosswind (90° and 270°) or downwind (180°) during the test period (Fig. 3C, D). Walking distances in the 90° angles were significantly different between the four directions during the test period (Kruskal-Wallis test, *P*<0.01), but were not significantly different during the control and end-control periods (Kruskal-Wallis test, α = 0.05) (Fig. 3C). Weevils walked a greater distance upwind during the test period compared with the control period; upwind (0°) index was significantly greater than zero (Wilcoxon signed rank test, *P*<0.005) (Fig. 3D). Orientation indices were significantly different between the four directions (Kruskal-Wallis test, *P*<0.01).

### Orientation Responses to Colored Lights and Volatiles

**Figure 4:**
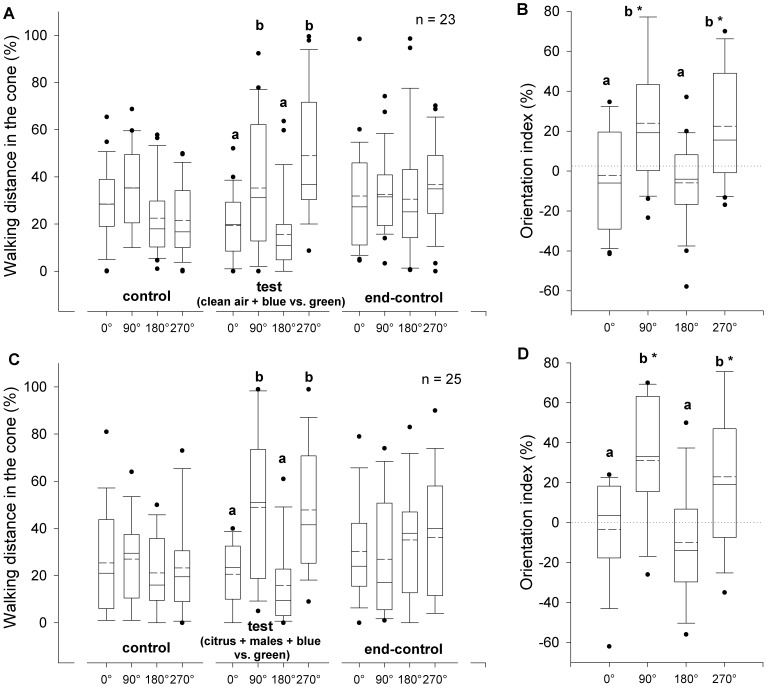
Orientation of weevils to volatiles produced by males feeding on citrus in the presence of colored lights. Orientation responses of *Diaprepes abbreviatus* adults, recorded during three consecutive 2-minute periods: **A.** clean air (control), clean air plus four opposite LEDs (test) and clean air again (end-control), or **C.** control, air passing through headspace of 10 male weevils feeding on citrus foliage plus four opposite LEDs (test) and end-control, were expressed as percentage of distance covered in each of four 90° angles projected to 0°, 90°, 180° and 270°. Orientation indices (**B.** and **D.** respectively) were calculated as the proportion of distance covered during the test period minus the proportion of the distance covered during the control period in each angle. Green LEDs were perpendicular to the air stream and blue LEDs were parallel to the air stream. Different letters indicate significant pairwise differences across the different directions (α = 0.05, Dunn’s post-hoc test). Asterisk indicates significance (α = 0.05, Wilcoxon signed rank test). In the boxplots, bold lines indicate medians, segmented line indicate the means, lower and upper boundaries of a box indicate the 25 and 75% quartiles, respectively, whiskers below and above the box indicate the 10th and 90th percentiles, respectively, and circles represent data beyond these limits (outliers).

When weevils were simultaneously stimulated with clean air plus two opposing blue LEDs parallel (0 and 180°) to the air stream versus two green LEDs perpendicular to the air stream during the test period (series III), individuals walked a greater distance crosswind (90° and 270° relative to the airflow) toward the green LEDs (Fig. 4A, B). Percentages of walking distance in the 90° angles were significantly different between the four directions during the test period (Kruskal-Wallis test, *P*<0.001), but were not significantly different during the control and end-control periods (Kruskal-Wallis test, α = 0.05) (Fig. 4A). Weevils walked a greater distance crosswind (90° and 270°) during the test period compared with the control period, i.e., crosswind indices were significantly greater than zero (Wilcoxon signed rank test, *P*< 0.001) (Fig. 4B). Orientation indices were significantly different between the four directions (Kruskal-Wallis test, *P*<0.001). Similar results were obtained when volatiles produced by 10 males feeding on citrus foliage were added to the air stream, and blue and green LEDs were positioned as in series III during the test period (series IV); individuals walked a greater distance crosswind (90° and 270°) toward the green LEDs (Fig. 4C, D). Percentages of walking distance in the 92° angles were significantly different between the four directions during the test period (Kruskal-Wallis test, *P*<0.001), but were not significantly different during the control and end-control periods (Kruskal-Wallis test, α = 0.05) (Fig. 4C). Weevils walked a greater distance crosswind (90° and 270°) during the test period compared with the previous control period, i.e., crosswind indices were significantly greater than zero (Wilcoxon signed rank test, *P*< 0.001) (Fig. 4D). Orientation indices were significantly different between the four directions (Kruskal-Wallis test, *P*<0.001).

### Kinematic Responses

Males walked faster (median = 25.7, IQR = 13.0 mm/s, *n = *110) than females (16.5, 14.5 mm/s, *n = *137) during experiments of group 1 and 2 (Wilcoxon unpaired test, *P* <10^−15^). The maximum speed recorded for males was 42.1 mm/s and for females was 39.2 mm/s.

In group 1 experiments, speed was affected by the different photic treatments (Kruskal-Wallis, α = 0.05); males walked at a lower speed (22.7, 9.7 mm/s, n = 6) in complete darkness compared with treatments where lights were present (28.4, 11.2 mm/s, *n* = 81) (Wilcoxon unpaired test, *P*<0.001), but females showed no difference in velocity between darkness (20.4, 15.4 mm/s, *n* = 6) and the other photic treatments (20.0, 10.1 mm/s, *n = *81).

**Figure 5:**
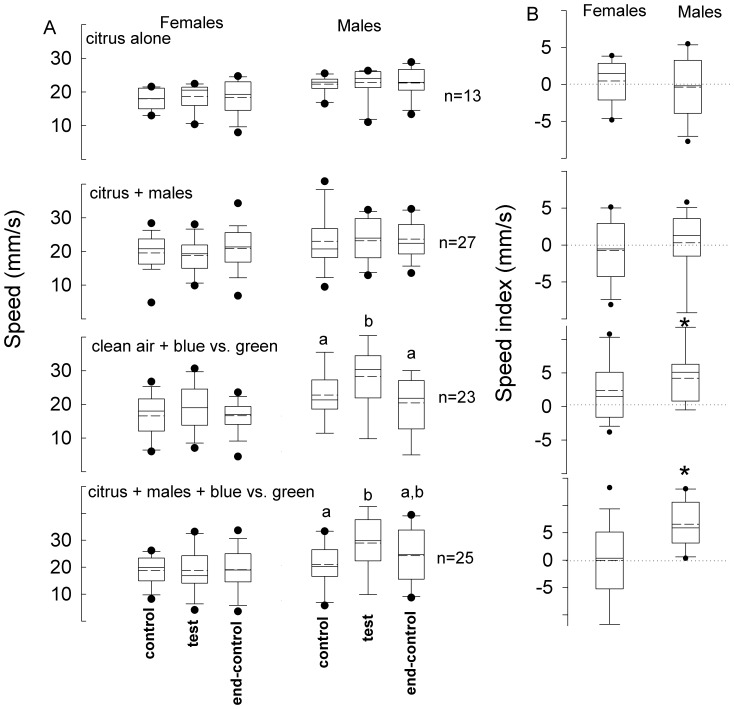
Walking speed of weevils elicited by different stimulus treatments. **A.** Average speeds of *Diaprepes abbreviatus* females and males, exposed to the different stimulation treatments , were recorded during the 3 three consecutive 2-minute periods (control, test and end-control). In the first series, weevils were stimulated during the test period with air that had passed over the headspace of citrus leaves (citrus alone) and in the second series, weevils were stimulated with air that had passed over male weevils feeding on citrus leaves (citrus + males). In a third experimental series, weevils were stimulated with colored LEDs during the test period; a blue LED was placed above the tube that served as source of clean air that stimulated the insect and another blue LED was placed on the opposite arm of this corridor, while the other two arms were provided with a green LED each (clean air + blue vs. green). Finally, each insect was given the choice between a blue LED + volatiles from males feeding on citrus versus another opposite blue LED and two green LEDs (citrus + males + blue vs. green); blue and green LEDs were positioned as in series III. **B.** Speed indices for each series were calculated as the average speed during the test period minus the average speed during the control period. Different letters indicate significant pairwise differences across the different directions (α = 0.05, Dunn’s post-hoc test). Asterisk indicates significance (α = 0.05, Wilcoxon signed rank test). In the boxplots, bold lines indicate medians, segmented line indicate the means, lower and upper boundaries of a box indicate the 25 and 75% quartiles, respectively, whiskers below and above the box indicate the 10th and 90th percentiles, respectively, and circles represent data beyond these limits (outliers).

In experiments of group 2, the addition of citrus volatiles (Fig. 5A, series I) and volatiles produced by 10 males feeding on citrus foliage (series II) to the air stream in the absence of light during the test period did not affect speed of the insects compared with their respective control and end-control periods (Kruskal-Wallis, α = 0.05). Insect speed indices associated with series I and II experiments were not significantly different from zero (Wilcoxon paired test, α = 0.05) (Fig. 5B). However, photic stimulation in the presence of clean air (Fig. 5A, series III) and photic stimulation + volatiles produced by 10 males feeding on citrus foliage (series IV) induced males (Kruskal-Wallis, *P*<0.005), but not females (Kruskal-Wallis, α = 0.05), to increase their speed during the test period compared with their respective previous control periods. Consistently, male speed indices for series III and IV experiments were significantly greater than zero (Wilcoxon paired test, α = 0.05) (Fig. 5B).

**Figure 6:**
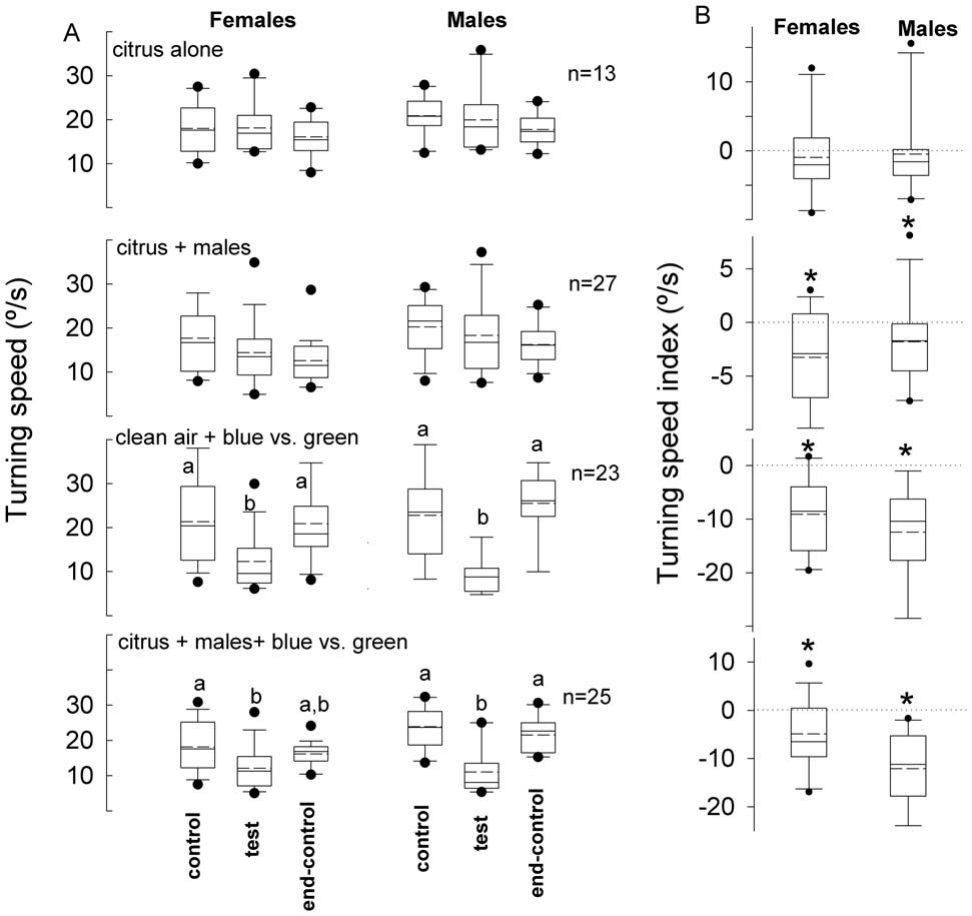
Turning speed of weevils elicited by different stimulus treatments. **A.** Average turning speeds of *Diaprepes abbreviatus* females and males, exposed to the different stimulus treatments , were recorded during the 3 three consecutive 2-minute periods (control, test and end-control). For treatments explanations see figure 5. **B.** Turning speed indices for each series were calculated as the average turning speed during the test period minus the average turning speed during the control period. Different letters indicate significant pairwise differences across the different directions (α = 0.05, Dunn’s post-hoc test). Asterisk indicates significance (α = 0.05, Wilcoxon signed rank test). In the boxplots, bold lines indicate medians, segmented line indicate the means, lower and upper boundaries of a box indicate the 25 and 75% quartiles, respectively, whiskers below and above the box indicate the 10th and 90th percentiles, respectively, and circles represent data beyond these limits (outliers).

Path straightness and turning speed were unaffected by the different photic treatments (Kruskal-Wallis, α = 0.05). The addition of citrus volatiles (Fig. 6A, series I) and volatiles produced by 10 males feeding on citrus foliage (series II) during the test period did not affect insect turning speed compared with the control and end-control periods (Kruskal-Wallis, α = 0.05). Turning speed indices were not significantly different from zero for series I (Wilcoxon paired test, α = 0.05) (Fig. 6B), but turning speed indices were significantly lower from zero for series II (Wilcoxon paired test, *P*<0.005). Photic stimulation in the presence of clean air (Fig. 6, series III) and photic stimulation + male-feeding produced volatiles (series IV) induced males (Kruskal-Wallis, *P*<0.001) and females (Kruskal-Wallis, *P*<0.001) to decrease their turning speed during the test period compared with the control and end-control periods. Consistently, turning speed indices were significantly less than zero for series III and IV (Wilcoxon paired test, *P*<0.001) (Fig. 6B). Males turned faster (21.80 ± 7.79°/s, *n = *110) than females (17.97 ± 7.10 mm/s, *n = *110) during all control periods of group 2 experiments (unpaired t-test, *P*<10^−5^), but such a difference was not observed when comparing males versus females in the test and end-control periods (unpaired t-test, *P* > 0.05).

**Figure 7:**
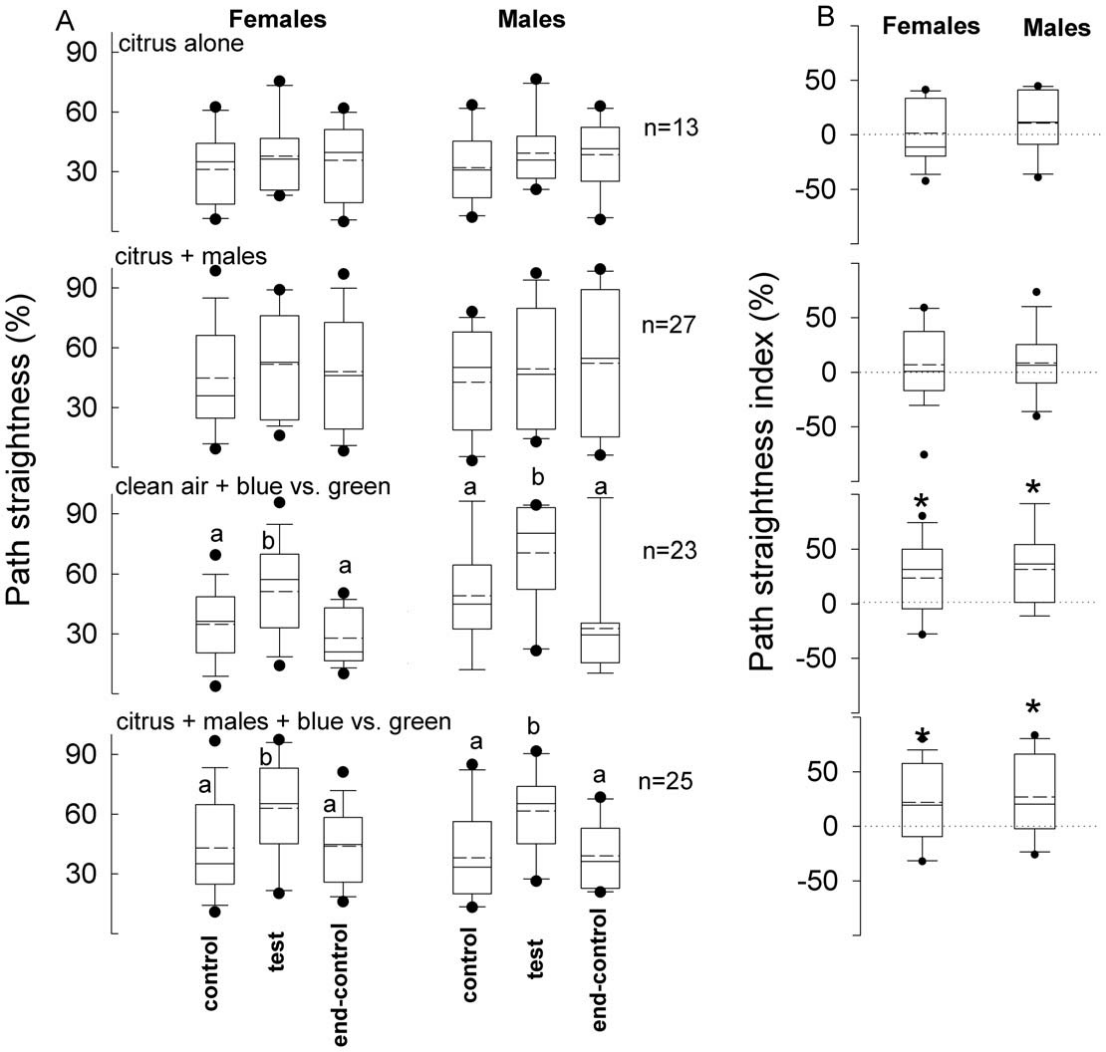
Path striaghtness of weevils elicited by different stimulus treatments. **A.** Path straightness of *Diaprepes abbreviatus* females and males, exposed to different stimulus treatments , were recorded during the 3 three consecutive 2-minute periods (control, test and end-control). For treatments explanations see figure 5. **B.** Path straightness indices for each series were calculated as the average path straightness during the test period minus the average path straightness during the control period. Different letters indicate significant pairwise differences across the different directions (α = 0.05, Dunn’s post-hoc test). Asterisk indicates significance (α = 0.05, Wilcoxon signed rank test). In the boxplots, bold lines indicate medians, segmented line indicate the means, lower and upper boundaries of a box indicate the 25 and 75% quartiles, respectively, whiskers below and above the box indicate the 10th and 90th percentiles, respectively, and circles represent data beyond these limits (outliers).

The addition of citrus volatiles (Fig. 7A, series I) and those released during feeding by males on citrus (series II) during the test period did not affect insect path straightness compared with the control and end-control periods (Kruskal-Wallis, α = 0.05). Insect speed indices for series III and IV were not significantly different than zero (Wilcoxon paired test, α = 0.05) (Fig. 7B). Photic stimulation in the presence of clean air (Fig. 7A, series III) and photic stimulation + male-feeding produced volatiles (series IV) induced males (Kruskal-Wallis, *P*<0.001) as well as females (Kruskal-Wallis, *P*<0.0001) to increase their path straightness during the test period compared with the control and end-control periods. Consistently, path straightness indices were significantly greater than zero for series III and IV (Wilcoxon paired test, *P*<0.01) (Fig. 7B). No difference was observed between female and male path straightness in group 1 and 2 experiments (unpaired t-test, *P* > 0.05).

## Discussion

The first group of experiments demonstrated a preference by *D. abbreviatus* for green versus blue or ultraviolet light, and no preference expressed when offered a choice of green or yellow. As the walls of the corridors were made of reflective material, it is possible that the LED lights could have been taken as background illumination by the insects. The adaptive value of phytophagous insects' preference for green and yellow wavelengths reflected by most plant foliage including citrus leaves [38] has been discussed in previous works [39], [16]. It is believed that attraction of herbivorous insects to green and yellow wavelengths allows them to discriminate foliage (reflectance range of 500 to 580 nm) from non-foliage hues (peaking at <500 nm or >580 nm) [39]. On the other hand, green light elicited less attraction than darkness or dimmed green light, i.e., individuals preferred to walk toward darkened corridors or those where light was lower. The fact that the weevils showed preference for red versus green or darkness suggests they may not perceive red or, perceive it as a dim light. Most insects are relatively insensitive to red as they do not have specific receptors responsive to this part of the spectrum [40]. This negative phototaxis was unexpected because *D. abbreviatus* usually exhibits a diurnal rhythm of activity, such that field populations feed and mate during photophase while oviposition occurs during scotophase [7]. The negative phototaxis observed here could also be associated with the search for refuge by disturbed individuals, suggesting a possible escape response. We cannot discard that this behavior could be associated with the fact that insects were induced to walk by touching a posterior leg with forceps.

Our first results allowed us to select green as an attractive color and blue as an unattractive control cue to be compared with a chemical attractant in the second (multimodal) set of experiments. When volatiles emanating from citrus alone or males feeding upon citrus were compared, the latter was the most potent attractant likely due to the presence of the male-produced aggregation pheromone, methyl (E)-3-(2-hydroxyethyl)-4-methyl-2-pentenoate [13]. Although weevils could be performing an escape response, the response induced by the chemosensory cue revealed a clear attraction behavior. In evoking attraction, it is probable that the pheromone played a key role by diminishing the repellency associated with insect stress and the emission of secondary metabolites (i.e., plant defense volatiles) commonly produced by foliage damaged by feeding. The combination of host volatiles + pheromones is known as a powerful attractant, likely mimicking conditions encountered by the insect [41], [42], [43]. Under the conditions of our study, green light was preferred over the chemical stimulus produced by conspecifics feeding on the host. We tested color preference in a dark arena in order to control other stimulus variables, thus assuring that the orientation response was evoked by the single light stimulus. The behavior of weevils, and the hierarchy observed between colors and scents could be different under other light conditions.

The multimodal stimulation study performed with Colorado potato beetle, *Leptinotarsa decemlineata,* in a similar dual-choice arena revealed subordination of chemotaxis to phototaxis [24]. Green and yellow were the most attractive lights for *L. decemlineata* and darkness was unattractive. *L. decemlineata* was positively phototactic when offered dark and light cues [24]. In contrast, *D. abbreviatus* did not show a strong phototactic response. Green light elicited less attraction than darkness or dim green light. Responses observed here suggest that hue, not brightness of the emitted green light, was mainly responsible for the attraction. For this reason, blue was selected as an unattractive cue to be offered versus green in the presence of the olfactory stimulus.

Kinematic parameters such as speed, turning speed and path straightness were affected by different stimulus treatments. The observed decreased turning speed and increased path straightness showed that weevils walked less tortuously in the presence of light emitted by LEDs during the test period compared with the control and end-control periods. Similarly, CPB turned at a lower rate and walked a straighter path in the presence of lights of different wavelengths compared with darkness [16]. The comparatively greater amount of information provided by vision presumably allows these insects locomotory control resulting in less tortuous paths in the presence of light. Volatiles produced by males feeding on citrus also induced a slight decrease of turning speed, highlighting the role of this odor as a guiding stimulus. In the absence of stimuli such as lights or an attractive odor, insects probably need to perform a faster sequential sampling to facilitate their searching, moving the antennal receptors of their bodies from one place to another in order to measure a temporal gradient that could allow them to infer the spatial gradient [15]. This maneuvering in which turns are oriented randomly with respect to the stimulus field in the absence of information about its target has been termed *klinokinesis* or *indirect guiding* [15]. Male weevilś increase in speed during photo-stimulation can be explained similarly; light provides spatial information that facilitates *direct guiding* wherein information is obtained about the orientation of the stimulus field resulting in reduced sampling. *Leptinotarsa decemlineata* also achieved higher speeds in the presence of light [24], a response that can be associated with direct guiding behavior.

It is unclear why female *D. abbreviatus* did not walk faster than males given their larger size and longer legs [25] as reported for female CPB [24]. Positive correlation of walking speed with size may only occur below a maximum size [44], [45]; females and males of this weevil species are larger than *L. decemlineata* [46], [47]. Alternatively, the presence of an innate response associated with some unknown fitness value could be involved.

Although several authors have performed similar multimodal studies, they have mostly focused on the *synergistic* effect between color and odor stimuli on choice behavior of phytophagous insects such as *Manduca sexta* (Lepidoptera: Sphingidae) [18], [21], *Homalodisca coagulata* (Homoptera: Cicadellidae) [22], *Bombus terrestris* (Hymenoptera: Apidae) [48] and *B. impatiens* [23]. Here, we demonstrated the disruptive effect of a colored light over odor stimuli, and measured kinematic parameters that were not taken into account by previous researchers. Orientation behavior similar to what we observed for *D. abbreviatus* was shown for *Trichoplusia ni* (Lepidoptera: Noctuidae) in a wind tunnel [17]. When male moths were simultaneously presented with a low-intensity incandescent light and a female-produced sex pheromone, more males congregated at the light than at the pheromone source. Primacy of visual over olfactory cues was also reported for *Vanessa indica* (Lepidoptera: Nymphalidae). Naïve butterflies depended primarily on color and secondarily on scent during flower visitation [19]. In dual-choice experiments, *M. sexta* moths showed a strong bias for a visual display over an odor plume, suggesting the former to be the primary attractant to a nectar source [21]. The diurnal moth *Macroglossum stellatarum* (Lepidoptera: Sphingidae) strongly favored a visual stimulus when presented simultaneously with an odor [20]. On the other hand, the nocturnal moth *Deilephila elpenor* (Lepidoptera: Sphingidae) responded preferably to odor [20]. Positive phototaxis in the beetle *Trypodendron lineatum* (Coleoptera: Scolytidae) prior to flight experience was abolished after beetles experienced flight coinciding with a switch to chemotactic orientation [49]. In contrast to flight-naïve *T. lineatum* that showed no chemotactic behavior regardless of the presence or absence of light, *D. abbreviatus* showed a hierarchy between chemo- and phototaxis in the fourth series of experiments presented herein, where the presence of the green light abolished upwind anemotaxis elicited by the combination of plant + male volatiles.

Many phytophagous insects use olfactory cues for long-range orientation to their host plants, and visual (and taste) cues at close range. Specific odors from the host plant combined with the pheromone probably are used by *D. abbreviatus* for long-range searching. Although, it is difficult to clarify if the responses observed here are long- or short-range, weevil preference of green color over the attractive odors suggests a short range response; the insect walking on the servosphere presumably senses that it is close enough to the plant because the odor intensity does not change while it walks, and uses green color to complete its search for food. This hypothesis would predict the results of this and previous studies. The adaptative value for the modulation of semiochemical attractiveness by vision, i.e., dominance of positive green-phototaxis over blue-phototaxis and chemotaxis as an orientating mechanism in *D. abbreviatus*, is not immediately obvious. However, insight into such strong responses to visual stimuli by chemically stimulated insects may be provided when the amount of information supplied by vision and olfaction is compared. The information transmission capacity of complex eyes is estimated to be several orders of magnitude higher than the olfactory system [15]. A broader discussion of this matter was presented elsewhere [24].

The results obtained in this study may be useful to develop applications to control this pest. These finding may serve in the design of a hypothetical trap for capturing weevil adults.
