# Correction: Olfactory Cues Are Subordinate to Visual Stimuli in a Neotropical Generalist Weevil

**DOI:** 10.1371/annotation/82488be5-8e90-4f03-97f4-5e1822b5623d

**Published:** 2013-09-19

**Authors:** Fernando Otálora-Luna, Stephen L. Lapointe, Joseph C. Dickens

There was an error in the first affiliation for author Fernando Otálora-Luna. The affiliation should be:

Laboratorio de Ecología Sensorial, Centro Multidisciplinario de Ciencias, Instituto Venezolano de Investigaciones Científicas (IVIC), Loma de Los Guamos, Parroquia Jají, Edo. Mérida, República Bolivariana de Venezuela 

